# Natural Autophagy Activators to Fight Age-Related Diseases

**DOI:** 10.3390/cells13191611

**Published:** 2024-09-26

**Authors:** Vianey M. Mundo Rivera, José Roberto Tlacuahuac Juárez, Nadia Mireya Murillo Melo, Norberto Leyva Garcia, Jonathan J. Magaña, Joaquín Cordero Martínez, Guadalupe Elizabeth Jiménez Gutierrez

**Affiliations:** 1 Departamento de Bioingeniería, Escuela de Ingeniería y Ciencias, Tecnologico de Monterrey, Campus Ciudad de México, Mexico City 14380, Mexico; a016532080@tec.mx (V.M.M.R.); nmurillo@inr.gob.mx (N.M.M.M.); magana.jj@tec.mx (J.J.M.); 2 Laboratorio de Bioquímica Farmacológica, Departamento de Bioquímica, Escuela Nacional de Ciencias Biológicas, Instituto Politécnico Nacional, Mexico City 11340, Mexico; jtlacuahuacj2300@alumno.ipn.mx; 3 Laboratorio de Medicina Genómica, Instituto Nacional de Rehabilitación Luis Guillermo Ibarra Ibarra, Mexico City 14389, Mexico; nleyva@inr.gob.mx

**Keywords:** autophagy, age-related diseases, natural compounds, cardiopathies, neurodegeneration, cataract

## Abstract

The constant increase in the elderly population presents significant challenges in addressing new social, economic, and health problems concerning this population. With respect to health, aging is a primary risk factor for age-related diseases, which are driven by interconnected molecular hallmarks that influence the development of these diseases. One of the main mechanisms that has attracted more attention to aging is autophagy, a catabolic process that removes and recycles damaged or dysfunctional cell components to preserve cell viability. The autophagy process can be induced or deregulated in response to a wide range of internal or external stimuli, such as starvation, oxidative stress, hypoxia, damaged organelles, infectious pathogens, and aging. Natural compounds that promote the stimulation of autophagy regulatory pathways, such as mTOR, FoxO1/3, AMPK, and Sirt1, lead to increased levels of essential proteins such as Beclin-1 and LC3, as well as a decrease in p62. These changes indicate the activation of autophagic flux, which is known to be decreased in cardiovascular diseases, neurodegeneration, and cataracts. The regulated administration of natural compounds offers an adjuvant therapeutic alternative in age-related diseases; however, more experimental evidence is needed to support and confirm these health benefits. Hence, this review aims to highlight the potential benefits of natural compounds in regulating autophagy pathways as an alternative approach to combating age-related diseases.

## 1. Introduction

The global elderly population is rapidly increasing. According to the United Nations’ 2023 World Social Report, the number of people aged 65 and older is projected to double from 761 million in 2021 to 1.6 billion by 2050 [1]. This demographic shift presents significant challenges in addressing new social, economic, and health problems concerning the elderly. From the perspective of health care, aging is the primary risk factor for various age-related diseases (ARDs), including cardiovascular, neurodegenerative, and metabolic diseases [2]. Given this trend, it is urgent to understand more about the complex interplay between the molecular mechanisms and environmental factors implicated in the aging process to promote healthier aging and an improvement in quality of life.

The phenomenon of aging is driven by various interconnected molecular hallmarks that influence the development of ARDs. Key factors such as loss of proteostasis, deregulated nutrient sensing, mitochondrial dysfunction, cellular senescence, and impaired autophagy are gaining attention due to their direct impact on the progression of ARDs [3,4,5]. Various strategies have been proposed to counteract these effects; however, significant challenges and uncertainties remain.

**Figure 1:**
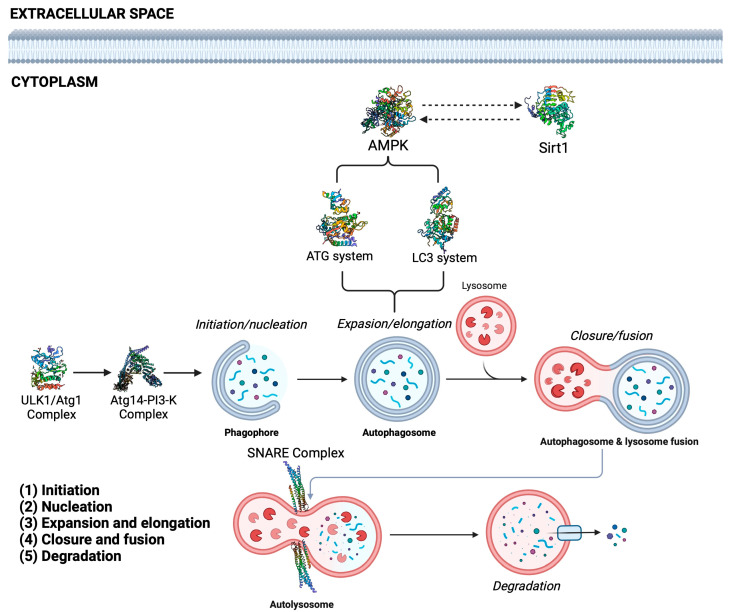
The autophagy process. The initiation stage involves the participation of protein complexes such as ULK-Atg1 (PDBID: 6QAS), Class III PI3-K complex ATG9, Rab1, TRAPPIII, and ubiquitin-like molecules (LC3A-D) (PDBID: 2Z0E). In the nucleation stage, the membrane begins to expand by recruiting proteins and lipids, transforming into a primary double membrane sequestering compartment structure called a phagophore. In this stage, the most important event is the recruiting of the proteins for the ATG14-Class III PI3-K complex (PDBID: 8SOR). During the expansion and elongation process, the membrane of the phagophore expands and closes around its cargo to form the autophagosome; also, the objective of this stage is to determine the site of lipidation of LC3. The closure and fusion stage is characterized by the maturation of the autophagosomes and their fusion with mature lysosomes to become autolysosomes, regulated by the SNARE complex (PDBID: 1KIL). Finally, in the degradation stage, macromolecules contained in the autolysosome are degraded by lysosomal hydrolases into monomeric units and returned to the cytoplasm to be recycled; in addition, the process is modulated by activation of signal pathways as AMPK (PDBID: 4CFF), which in turn is modulated by Sirt1 (PDBID: 5BTR).

One of the main mechanisms that has attracted more attention to aging is autophagy, a key catabolic process to remove and recycle damaged or dysfunctional cell components (protein aggregates, damaged mitochondria, peroxisomes, ribosomes, endoplasmic reticulum, endosomes, lipid droplets, and intracellular pathogens) [6] to support energy needs and maintain cellular homeostasis in order to preserve cell viability [7]. The three principal types of autophagy on eukaryotic cells are macroautophagy, microautophagy, and chaperone-mediated autophagy [8,9]. The macroautophagy process is described as the formation of the autophagosome and is distinguished by the involvement of the autophagy-related proteins (Atg) [10]. Microautophagy describes the direct translocation of cytosolic material into lysosomes via membrane invagination in order to contribute to cellular homeostasis [10,11]. In contrast, chaperone-mediated autophagy shows specificity, using only proteins as a target. This process does not depend on the autophagosome formation; therefore, the proteins are directly translocated into the lysosomes for degradation [12,13]. Macroautophagy is divided into five stages: (1) initiation, (2) nucleation, (3) expansion and elongation, (4) closure and fusion, and (5) degradation (Figure 1) [14]. The initiation stage involves the participation of protein complexes such as ULK-Atg1, Class III PI3-K complex, ATG9, Rab1, TRAPPIII, and ubiquitin-like molecules (LC3A-D), and is characterized by the formation of the omegasome, which is an enriched endoplastic reticulum domain with phosphatidylinositol-3-phosphate. The omegasome will serve as an initiation site for the formation of the phagophore, which is the membrane precursor of the autophagosome, and is generated at multiple sites throughout the cytoplasm [6,15]. In the nucleation stage, the membrane begins to expand by recruiting proteins and lipids, transforming into a primary double membrane sequestering compartment structure named the phagophore [16]. In this stage, the most important event is the recruiting of the proteins for the ATG14-Class III/PI3-K complex. This complex produces the phosphatidylinositol-3-phosphate needed for macroautophagy and for the recruiting of vacuolar protein sorting VPS15 and VPS34, as well as the release of Beclin1 and AMBRA1 from their inhibitor Bcl-2 [17]. During the expansion and elongation process, the membrane of the phagophore expands and closes around its cargo to form the autophagosome [16]. Also, the objective of this stage is to determine the site of lipidation of LC3, which is determined by the activity of the elongation complex ATG5-12/16L1. LC3 lipidation (LC3-II) is required for its association with the autophagosomal membrane; therefore, the membrane-associated LC3 will allow completion of the autophagosomal formation, also interacting with p62/SQSTM1 inside the autophagosome [18,19,20]. The closure and fusion stage are characterized by the maturation of the autophagosomes and their fusion with mature lysosomes to become autolysosomes [21]. The process is regulated by SNARE proteins and STX17, which colocalizes to complete the autophagosome formation; also, STX17 interacts with SNAP29 and lysosomal VAMP8, leading to the autophagosome–lysosome complete fusion [22]. Finally, in the degradation stage, macromolecules contained in the autolysosome are degraded by lysosomal hydrolases into monomeric units and returned to the cytoplasm to be recycled (Figure 1) [23].

The autophagy process can be induced, impeded, or deregulated in response to a wide range of internal or external stimuli [24], such as starvation, oxidative stress, hypoxia, damaged organelles, the presence of infectious pathogens, and aging [25]. Autophagy is known to decline with age, directly contributing to impaired protein homeostasis [26] that in turn leads to protein aggregates and dysfunctional protein turnover, which are molecular hallmarks of aging and increase the incidence of age-related diseases (ARDs) [27,28].

In this review, we provide an update about the role of natural compounds in the modulation of autophagy in common age-related diseases.

## 2. Cardiopathies

Cardiac diseases are among the most common ARDs and one of the top three causes of death worldwide. It is expected that cardiac diseases will impose a burden on the global economy and health care systems [2]. Cardiomyopathies are characterized by the suppression of autophagy in cardiac cells, which plays a crucial role in maintaining cardiac homeostasis. The disruption of this process is associated with various cardiac conditions such as cardiac hypertrophy, myocardial infarction, diabetic cardiomyopathy, and ultimately, cardiac failure [29].

Human aging is typically accompanied by cardiac hypertrophic remodeling and a progressive decline of left ventricular (LV) diastolic function [30,31]. In addition, cardiomyocytes, the essential cellular constituents of the cardiovascular system, are primarily found in a post-mitotic state, causing them to heavily depend on intact autophagy and mitophagy to preserve their physiological functions [30]. As we age, the cardiovascular system’s efficiency declines. To combat this, autophagy plays a crucial role in promoting the survival of dormant cells within the cardiovascular system and counteracting cell death caused by apoptosis or necrosis following injury [30].

The molecular mechanism of cardiovascular diseases includes two essential pathways: metabolic factors and mutations in cardiovascular disease-related sites. This section focuses on signaling cascades of PI3K/AKT/mTOR, epidermal growth factor (EGFR), insulin-like growth factor (IGF), AMPK/mTOR, and mitogen-activated protein kinases (MAPKs), among others [32]. Focusing on autophagy, the AMPK/mTOR signaling pathway plays an essential role in autophagy regulation, cell growth, cell proliferation, and metabolism. Several studies have shown that this signaling pathway participates in the process of cardiovascular diseases [32]. On the other hand, the silent information regulator 1 (Sirt1) also exerts a cardioprotective effect in various cardiovascular diseases via multiple cell activities [33].

Previous evidence shows that cells cope with environmental stress by targeting abundant transcription factors, such as the Forkhead box O (FoxO) proteins, which play a critical role in the cardiac pathologies associated with aging by regulating both metabolic and proliferative events in endothelial cells [34]. Indeed, a study by Hariharan et al. in 2010 demonstrated that Sirt1 mediates glucose starvation-induced autophagy by deacetylating FoxO in cardiomyocytes [35]. Additionally, Sirt1 overexpression protects the heart from ischemia-reperfusion injury by inhibiting proapoptotic molecules, demonstrating that Sirt1 is involved in cardio protection.

Next, we highlight some natural compounds known to induce autophagy and that have been reported to offer beneficial effects for age-related heart diseases.

### 2.1. Curcumin

Curcumin (1,7-bis-(4-hydroxy-3-methoxyphenyl) is the main biocompound that comes from the spice turmeric, and it has been widely used in Indian and Chinese traditional medicine because of its diverse biological benefits, such as anti-inflammatory, antioxidant, and anticancer properties [36,37,38,39]. Research has also highlighted curcumin’s ability to induce autophagy, particularly in cancer cells, where autophagy is a precursor to apoptosis [40,41,42]. This process is mediated through the AMPK signaling pathway and the activation of transcription factor EB (TFEB), both of which positively regulate autophagy [43,44]. Research on the effect of curcumin on autophagy has explored its effect on lifespan in models like C. elegans, D. melanogaster, yeast, and even mice, showing influence on pathways like mTOR, PKA, and FoxO [45,46,47,48]. Furthermore, studies made on various diseases (chronic kidney disease and insulin resistance in non-alcoholic fatty liver disease), demonstrated that the administration of this compound increased the ATP levels and inhibited their depletion and ameliorated mitochondrial dysfunction [49,50,51].

**Figure 2:**
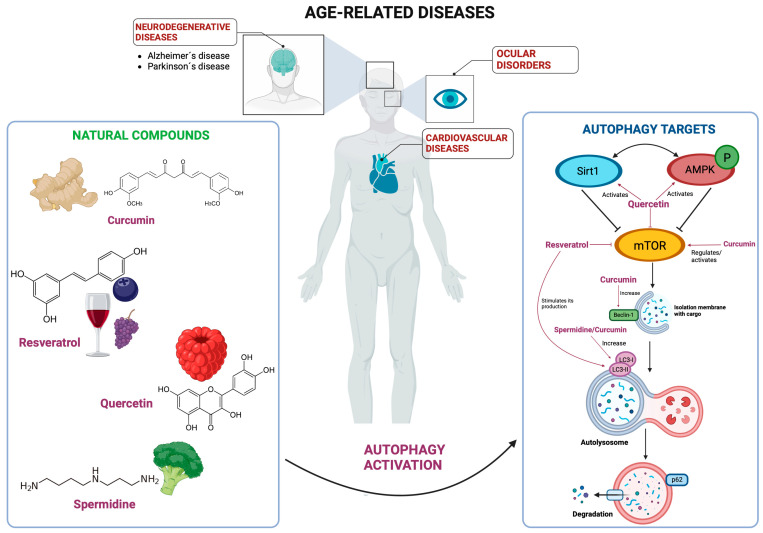
Natural autophagy activators in age-related diseases. On the left: natural compounds (curcumin, resveratrol, quercetin, and polyamines such as spermidine) that can induce autophagy in the context of age-related diseases. On the right: main regulatory targets of autophagy that, when activated, enhance autophagic flux, which is often diminished in cardiovascular diseases, neurodegenerative disorders, and cataracts.

Evidence has highlighted curcumin as a molecule that activates autophagic flux and attenuates oxidative damage via AKT/mTOR pathway regulation, as shown by research in diabetic rats with myocardial damage, where curcumin treatment in combination with high intensity interval training (HIIT) demonstrated improvement in the expression of important autophagic proteins such as ATG-5 and Beclin-1 [52]. In mice with doxorubicin-induced cardiomyopathy, where curcumin attenuated oxidative stress and inflammation, curcumin also restored the alterations in autophagy, as shown by increased levels of Beclin-1 and of the LC3II/LC3I ratio and by activation of the AKT/mTOR pathway [53]. More evidence extrapolates the benefits of curcumin on reducing senescent cardiomyocytes via AMPK/mTOR in a dose-dependent manner, which suggests the role of curcumin as an anti-cardiac aging molecule (Figure 2) [54]. All of this evidence demonstrates the advantages of curcumin administration on cardiac homeostasis via autophagy activation. It is worth noting that the Food and Drug Administration (FDA) has not approved the use of curcumin for any medical condition; however, it is available in the United States as a dietary supplement [55].

### 2.2. Resveratrol

Resveratrol (3,5,4-trihydroxystilbene) is a natural polyphenol produced by various plants and fruits in response to external stimuli. It is commonly found in peanuts, berries, grapes, cranberries, and other products, such as red wine [56]. In the last decades, resveratrol has attracted much attention due to its beneficial properties. It has been shown to decrease oxidative stress through upregulation of antioxidant enzymes like catalase (CAT) (EC 1.11.1.6) and superoxide dismutase (SOD) (EC 1.15.1.1) [57,58]. Moreover, its anti-inflammatory effects occur through the modulation of Sirt1, which in turn inhibits the NF-κB/STAT pathway, thereby reducing the production of inflammatory molecules [59]. Additionally, resveratrol modulates diverse pathways that converge in cancer, such as Raf/MAK, ERK/p-38, and PI3K-AKT, among others [60].

Much evidence about resveratrol has pointed to a direct relationship with autophagy induction via inhibition of the mTOR-ULK1 pathway and stimulation of LC3-II production [61,62]. Moreover, the effects of resveratrol were studied in mitochondrial dysfunction and oxidative stress, and the results showed that a resveratrol treatment increased citrate synthase activity, basal oxygen consumption, and mitochondrial ATP production [63]. In line with this, resveratrol has been demonstrated to be a good candidate to fight ARDs.

In recent years, natural compounds with cardioprotective properties have been widely studied [64,65], and resveratrol is no exception. In studies in vivo on diabetic rats with cardiomyopathy and in vitro assays using neonatal rat ventricular myocytes, resveratrol has shown to negatively regulate the PI3K/Akt pathway in order to stimulate autophagic flux, decreasing apoptosis and ameliorating cardiac dysfunction [66]. Nevertheless, the effects of resveratrol also extrapolate to other pathways that converge in autophagy activation. For example, through the activation of the master autophagy regulator TFEB, resveratrol lessens endothelial oxidative damage of cardiovascular models [67]. Additionally, the activation of Sirt1 directly helps to increase the expression of autophagic molecules such as LC3 and Beclin-1 (Figure 2) [68]. This evidence suggests that the use of resveratrol could help to improve health in cardiomyopathies.

### 2.3. Quercetin

Quercetin (2-(3,4-dihydroxyphenyl)-5,7-dihydroxy-4H-1-benzopyran-4-one) is one of the most common flavonoids present in fruits like grapes, berries, cherries and other citric fruits, in vegetables like onions and broccoli, and in seeds and other plants [69,70]. Antioxidant and anti-inflammatory properties are the most studied in quercetin [71]. Research has also highlighted its potential as an anti-diabetic and anti-carcinogenic agent [72,73,74,75]. Interestingly, quercetin has also been shown to stimulate autophagy via signaling pathways that converge in the inhibition of mTOR through the activation of AMPK and Sirt1, ultimately leading to an increase in the LC3-II/LC3-I ratio [76,77]. Additionally, quercetin was used to treat lipopolysaccharide-induced oxidative damage, and the results showed that quercetin improved ATP levels, leading to a beneficial effect in mitochondrial function [78].

In recent years, quercetin has attracted attention to cardiovascular diseases due to its demonstrated effect on autophagic stimulation. Specifically, quercetin treatment has been shown to ameliorate myocardial injury in diabetic rats through the activation of the AMPK pathway, leading to increased LC3 and Beclin-1 protein levels and amelioration of oxidative stress [79]. Furthermore, quercetin has been shown to suppress the PI3K/Akt pathway that results in autophagy activation and attenuation of myocardial ischemia in Sprague-Dawley rats [80]. Also, quercetin has been shown to promote autophagy by increasing the LC3 I/II ratio, improving endothelial function over time and decreasing oxidative stress to attenuate hypertension in Wistar rats [81]. Evidence has shown that quercetin prevents myocardial fibrosis via the modulation of miR-223-3p/FOXO3, which promotes the regulation of the autophagic effector enzyme ATG7 that, in turn, increases LC3 levels. This research was performed on cardiac tissues of patients with atrial fibrillation and in aged rats [82].

All of these research findings highlight the beneficial autophagic effects of quercetin’s function as a promising adjuvant to prevent and treat age-related cardiopathies (Figure 2).

### 2.4. Polyamines

Polyamines (PAs) are small aliphatic molecules containing amino groups throughout their structure. They can be found in a wide range of foods, including fruits and vegetables with a higher content of polyamines, such as mushrooms, green peppers, green peas, and citrus fruits [83]. At physiological pH, these amino groups become protonated and acquire the capacity to interact with negatively charged biomolecules such as nucleic acids, proteins, and phospholipids [84,85,86]. The principal PAs in mammalian cells are putrescine, spermidine, and spermine; they are interconverted through a stepwise process, typically starting with arginine [87]. Deregulation of PAs, in particular spermidine and spermine, is related to cellular senescence, impaired proteostasis, dysregulated nutrient sensing, dysfunctional mitochondria, and dysfunctional autophagy [88]. Spermidine is mechanically related to autophagy activation through the posttranslational modification of the eIF5A, which, in turn, promotes the translation and synthesis of TFEB, a master autophagy regulator. Spermidine also regulates cell viability by significantly increasing cell metabolic activity and ATP production in a mitophagy deficit induced by disease-associated tau protein and cardiac aging [89,90].

Moreover, spermidine inhibits the histone acetyltransferase P300 (EP300) to promote the activation of various autophagy-related genes [91]. Additionally, spermidine inhibits Beclin-1 degradation to promote autophagy initiation [92].

Spermidine and spermine have been shown to exert cardioprotective effects on aged rats by preventing myocardial fibrosis and morphological myocardial alterations [93]; similar effects were also reported in aged mice where spermidine stimulated autophagy [31]. Furthermore, spermidine limits atherosclerotic lipidic plaque formation by inducing autophagy, as shown by the increase in the LC3 II/I ratio of mouse aortic vascular smooth muscle cells [94]. Additionally, spermidine has been demonstrated to ameliorate myocardial injury and cardiac dysfunction in mice by promoting autophagy mediated by the AMPK/mTOR pathway (Figure 2) [95]. All of this evidence makes spermidine a good candidate to prevent and ameliorate cardiovascular diseases.

## 3. Age-Related Neurodegenerative Diseases: Alzheimer’s and Parkinson’s

Aging is the primary risk factor for most neurodegenerative diseases, including Alzheimer’s disease (AD) and Parkinson’s disease (PD) [96]. One in ten individuals aged ≥65 years has AD, and its prevalence increases with age. Meanwhile, more than 10 million people worldwide are living with PD; additionally, older men are 1.5 times more likely to have PD than women. In both diseases, pathogenesis presents similar features, such as oxidative stress, mitochondrial dysfunction, and protein aggregation, all of which are events related to autophagy [96,97,98].

Autophagy is an essential process in preserving the homeostatic requirements of post-mitotic neurons [97]; it cooperates with the ubiquitin-proteasome system (UPS) to degrade toxic proteins, and its deficiency causes the accumulation of ubiquitinated proteins, axon dystrophy, abnormal synaptic transmission, and subsequent degeneration [99]. Furthermore, autophagic responses extinguish neuroinflammatory reactions, which directly contribute to the pathogenesis of PD and AD [30]. Moreover, autophagy is associated with other signaling pathways such as AMPK, mTOR, and Sirt1. Human studies suggest that the mTOR signaling pathway is involved in AD, as it is inhibited in the cortex and hippocampus of adult mice with the disease [100,101]. In PD, the accumulation of alpha-synuclein is known to play a key role in the death of dopamine-producing neurons. Therefore, enhancing autophagy, particularly through the mTOR signaling pathway, may help prevent the toxic accumulation of alpha-synuclein [102]. Autophagy-enhancing effects have been linked to Sirt1, which aids in the degradation of Aβ and promotes synaptic formation and synaptic activity. Therefore, Sirt1 can reduce the progression of various degenerative diseases [103,104]. Sirt1 plays an essential role in Aβ and tau metabolism through the deacetylation of site-specific lysine residues of histone and non-histone proteins [104]. As shown, the inhibition of Sirt1, or its decline in enzymatic activity observed with age, results in a reduction in the deacetylation of tau. This reduction might block tau polyubiquitination and tau proteasomal degradation, leading to p-tau accumulation. Consequently, this accumulation can result in the formation of neurofibrillary tangles (NFTs) [104]. Also, genetic variation in Sirt1 is linked with the development of PD along with genetic mutations in PD, which have been reported to alter the expression of the Sirt1 promoter with a subsequent reduction of the neuroprotective role of Sirt1 [103].

### 3.1. Curcumin

Regarding age-related neurodegenerative diseases, evidence indicates that oral intake of curcumin has beneficial effects on conditions like AD by preventing short-term memory deterioration [105]. Moreover, curcumin has been shown to reverse cognitive disorders in an AD mice model induced by cisplatin. This effect is achieved through the activation of the AMPK-JNK signaling pathway to inhibit mTOR and Bcl-2 that, in turn, modulate autophagy as demonstrated by the overexpression of LC3 and downregulation of p62 [106]. The beneficial effects of curcumin on autophagy extend to PD. In mouse models, curcumin treatment led to an increased expression of LC3-II, facilitating enhanced clearance of α-synuclein proteins. Notably, this was accompanied by improvements in mitochondrial function (Figure 2) [107]. Collectively, these findings underscore curcumin’s potential as a therapeutic candidate for various neurodegenerative diseases.

### 3.2. Resveratrol

Among neurodegenerative diseases, the role of resveratrol has been widely studied in AD due to its effect on pathways that converge on autophagy activation [108]. New research on animal models has shown that resveratrol enhances cognitive function in rats by stimulating the AKT/mTOR pathway [109]. Moreover, in Wistar rats with AD induced by colchicine, the combination of resveratrol and donepezil significantly increased the number of astrocytes and decreased microglia. This suggests that activating the Sirt1 pathway enhances mitophagy, leading to improved neuroprotection [110]. In the Drosophila model that overexpresses the amyloid precursor protein (APP) for a similar AD phenotype, resveratrol treatment showed improvements in sleep and memory that were dependent on the ortholog Sir2, a sirtuin ortholog that in turn stimulates autophagy by activating HSP70 and ATG4, also interacting to LC3 and p62 [111,112]. Meanwhile, a randomized trial of people affected by AD who orally consumed 500 mg of resveratrol once a day reported good tolerability. Notably, its principal metabolites can penetrate the blood-brain barrier, implying potential beneficial effects on the central nervous system (CNS) [113]. Benefits of resveratrol have also been described in PD; studies in mice models showed that resveratrol ameliorates the deficit in cognitive and motor function in a dose-dependent manner; moreover, the effects of resveratrol also demonstrated a decrease in α-synuclein aggregation [114]. A study using state-of-the-art hydrogen/deuterium exchange mass spectrometry technology demonstrated that resveratrol interferes with amyloid aggregation [115]. The effects of autophagy and resveratrol in PD were demonstrated in a 1-methyl-4-phenyl-1,2,3,6-tetrahydropyridine (MPTP)-induced mouse model of the disease, where LC3 promoted autophagic degradation of α-synuclein via the activation of the Sirt1 pathway (Figure 2) [116]. More recently, in two cell models of PD (SH-Sy5Y and SK-N-SH cell lines treated with MPP+ (1-Methyl-4-phenylpyridium)), resveratrol promoted autophagy as shown by the increase of the protein expression of Beclin-1 and LC3-II/LC3-I ratio, as well as the reduction in p62 levels; interestingly, the effects of resveratrol also reduced the expression of the lncRNA SNHG1 (small nucleolar RNA host gene 1), which positively regulates the SNCA gene that encodes for α-synuclein [117].

All of this evidence suggests that resveratrol is a promising natural compound with significant properties for the clearance of amyloid aggregates through autophagy stimulation.

### 3.3. Quercetin

Quercetin holds promise as a mitigator for neurodegenerative diseases. It activates Sirt1, a crucial regulator that influences the expression of key genes like FOXO1 and NRF2. These genes play interconnected roles in the autophagy pathway, suggesting quercetin’s potential to support neurological health through these regulatory mechanisms [118,119].

Recent research into the therapeutic potential of quercetin for AD has unveiled a whole network of molecular targets, with alpha serine/threonine-protein kinase-1 (AKT1) prominently featured (Figure 2). AKT1 is known to interact with Atg7, a critical enzyme that regulates the Atg proteins necessary to initiate autophagy [120,121]. Additionally, other research has shown that quercetin reduces the expression of Abelson kinase (Abl kinase), a significant molecule implicated in Alzheimer’s disease pathogenesis. By decreasing the expression of Abl kinase, quercetin helps to prevent tau phosphorylation and neuronal apoptosis triggered by Aβ, primarily through activation of the autophagy process [122,123]. Quercetin modified with Palladium nanomaterial (PdNPs) coated on polysorbate 80 has been shown to enhance autophagy and facilitate the clearance of Aβ in SH-SY5Y cells, a model for AD [124]. Furthermore, research on a PC12 cell model of AD has indicated that quercetin treatment improves cell survival and proliferation, mitigates Aβ toxicity, and enhances the expression of Sirt1 [125,126]. Moreover, evidence has shown that quercetin ameliorates neuronal death, mitochondrial dysfunction, and α-synuclein accumulation in animal and diverse cell models of PD by activating autophagy [127]. This effect is demonstrated by increased levels of ULK1(Ser555)/ULK1, p-TBK1(Ser172)/TBK1, and LC3 [128]. Furthermore, in a rotenone-induced rat model, quercetin treatment not only increased autophagy but also attenuated behavioral impairments [129].

### 3.4. Polyamines

Polyamines are crucial for central nervous system (CNS) function; among their different roles, they modulate N-methyl-D-aspartate (NMDA) receptors that, in turn, regulate diverse neurological functions, including memory [130,131]. Additionally, they promote the regulation of glutamate signaling, which also enhances excitability and memory [130].

It has long been known that PA levels are disrupted in the human brain with AD [132]; in fact, other research on mice models of the disease have demonstrated altered polyamine levels, as shown by increased anabolism and upregulation of spermidine synthase (EC 2.5.1.16) and elevated acetylspermidine levels, suggesting a breakdown issue [133]. Notably, the distinction between these acetylated polyamine forms and biogenic polyamines has a differential impact on tau protein accumulation [90].

Research suggests that oral supplementation of spermidine and spermine promotes autophagy in the hippocampus, thereby delaying brain aging and maintaining cognitive function in mice [134]. Additionally, evidence has shown the benefits of spermidine treatment on mitochondrial impairment through autophagy activation, as seen by elevated LC3 and Beclin levels compared to the control in SY5Y cells stably expressing a mutant form of human tau protein [90]. Research using high-resolution imaging demonstrated that spermidine could induce autophagy and clear amyloid precursor protein clusters, a key pathological feature of Alzheimer’s disease [135]. These findings suggest that spermidine, by promoting autophagy and restoring polyamine homeostasis, may hold promise as a therapeutic strategy for Alzheimer’s disease.

## 4. Age-Related Ocular Diseases: Cataracts

Visual impairments are among the leading diseases in developed countries, and aging is the primary cause of their clinical manifestation [30]. Cataracts are the most common cause of visual impairment in the elderly [136], and it is estimated that from 2000 to 2020, 43.3 million people went blind due to cataracts and 295 million had moderate to severe vision impairment (MSVI) due to this condition [137].

Age-related cataracts form in the center of the lens, which stiffens as new layers of cortical fibers continue to proliferate. In this condition, autophagy plays a crucial role in maintaining cell integrity and preserving the lens’s physical properties by ensuring cellular homeostasis and the absence of fiber accumulation [138,139,140]. Additionally, the autophagic role in the eye is highlighted by the high expression of autophagy-related proteins in various eye structures, including the cornea, lens, retina, and orbit. This protein expression correlates with significant autophagic activity observed particularly within the lens [138,141].

Studies on mice revealed the impact of altered autophagic flux mediated by TBC1D20, a key regulator of autophagosome maturation. The mice exhibited cataracts in the lens nucleus, along with highly disorganized, degenerated lens fibers and an accumulation of ubiquitin aggregates. These findings strongly suggest that autophagy is not only crucial for maintaining intracellular homeostasis in the lens but also that the accumulation of residual material contributes to cataract formation [138,142]. Multiple signaling molecules and pathways, such as mTOR and AMPK, play significant roles in the development of cataracts. For instance, the mTOR pathway influences cataract development by modulating various cellular processes, such as cell growth, autophagy, and epithelial-mesenchymal transition.

Similarly, the AMPK pathway is central in the pathogenesis of ocular diseases, affecting various ocular tissues. Studies have linked age-related inactivation of AMPK and autophagy to lens epithelial cell senescence and age-related cataracts [143,144]. Furthermore, Sirt1 has been detected in multiple eye structures in both mice and humans, including the cornea, lens, ciliary body, retinal pigment epithelium (RPE), neuroretina, and melanocyte. Numerous studies have also linked Sirt1 dysfunction to ocular diseases, including cataracts and age-associated macular degeneration [145]. Notably, Sirt1 levels in the lens were significantly decreased in individuals aged ≥51 years, further highlighting its potential role in cataract formation due to its widespread distribution in different eye cell types.

### 4.1. Curcumin

Curcumin has been demonstrated to have protective effects against cataract development and its progression in numerous in vitro and in vivo cataract models. In an in vitro study, it was shown that curcumin inhibited a pleiotropic oxidative stress response in cultured lens epithelial cells (hLECs) [146]. In another study performed in rats, curcumin suppressed selenium-induced oxidative stress in the rats’ lenses and delayed the formation of cataracts by inhibiting non-enzymatic antioxidant depletion [146,147]. The above suggests that the anti-cataract effect of curcumin may originate from its antioxidant properties.

As it concerns autophagy, a study performed in RPE cells demonstrated that curcumin, compared with rapamycin, exerts a similar amount of neuroprotection [148]. Recent research on rats with glucose-induced cataracts highlights the potential significance of tetrahydrocurcumin (THC), a principal metabolite of curcumin, and a synthetic curcumin analog (C1) in promoting lysosomal biogenesis and activating TFEB, consequently mitigating cataract formation [149]. These studies offer a new perspective on age-related cataracts, suggesting that curcumin may safely stimulate autophagy and could be a promising treatment option.

### 4.2. Resveratrol

While much of the research has primarily emphasized the anti-inflammatory advantages of resveratrol in addressing various age-related eye conditions, its autophagic properties have also shown promise in treating other ocular diseases, including age-related macular degeneration (AMD) [150], diabetic retinopathy [151], glaucoma [152], and cataracts. In fact, resveratrol emerges as a good candidate in the prevention and treatment of cataracts. Evidence indicates that resveratrol promotes the phosphorylation of p38, which in turn mitigates oxidative damage induced by high levels of glucose in human lens epithelial cells (HLEC) by triggering autophagy, as evidenced by the increased protein levels of LC3 and Beclin-1 (Figure 2) [153]. These autophagic effects have also been observed in the ARPE-19 cell line [154]. Also, research has shown that resveratrol and three of its main metabolites (resveratrol-3-O-glucuronide, resveratrol-4′-O-glucuronide, and resveratrol-3-O-sulfate) can be measured in different eye tissues, such as the aqueous humor, vitreous humor, and conjunctiva, suggesting the feasibility of using dosage-effective resveratrol treatment for ocular diseases [155].

This evidence supports the idea that autophagic stimulation by resveratrol could be applied to treat age-related ocular diseases, including cataracts.

### 4.3. Quercetin

The effects of quercetin on autophagy in ocular diseases are still being explored, especially in cataracts. However, new research has demonstrated that quercetin activates the Sirt1/AMK signaling pathway, a positive regulator of autophagy (Figure 2) [77]. Additionally, research has shown that quercetin decreases oxidative species by promoting autophagy through the NRF2-PGC-1α-Sirt1 pathway in mice with macular degeneration [156].

Quercetin and its metabolites can protect the lens from the internal flow of calcium and sodium, thereby maintaining the lens’s ion balance and protein levels, crucial for lens transparency [157]. Moreover, quercetin stimulates the hypoxia-inducible factor-1 (HIF1) pathway in lens epithelial cells, activating and synthesizing downstream effectors and thereby potentially delaying cataract progression [157].

Extensive in vitro and in vivo studies in animal models have consistently demonstrated the lens-protective effects of quercetin’s antioxidant and chelating properties [157,158,159]. In a rat model of cataracts induced by sodium selenite, the antioxidant properties of quercetin significantly reduced crystal turbidity and prevented the formation of cataracts caused by selenite [160]. It also bolstered the antioxidant mechanism of the lens, safeguarding its integrity. Furthermore, quercetin has shown promising results in protecting against cataracts induced by H_2_O_2_ (causing lens opacification) and retinal lesions induced by diabetes [161]. This evidence strongly suggests quercetin as a potential therapeutic target for cataracts.

It is also important to highlight quercetin as an alternative in treating other specific ocular diseases due to its proven antioxidant, anti-inflammatory, antifibrosis, and pro-autophagic properties. Its antioxidant effects could significantly impact the management of conditions besides cataracts, such as dry eye disease and retinopathy, among others [157].

### 4.4. Polyamines

Although direct studies on the autophagic role of spermidine in age-related cataracts have not been conducted, there is indirect evidence regarding its positive regulation of Sirt1 [88,162]. This regulatory role of spermidine should be considered crucial in the development of the disease because the downregulation of Sirt1 in age-related cataracts has been described in research [163,164].

It is also important to mention that there is evidence on the anti-aging and protective properties of PA in treating ocular diseases [165], particularly in cataracts. Research indicates that PAs are detectable in intraocular fluid [166], and a decrease in its levels is linked to the development of age-related cataracts [167]. Furthermore, studies in mice and rabbits with cataracts have shown a remarkable decrease in the level of spermidine [168,169]. This decrease is likely caused by increased catabolism activity and transglutaminase type 2 (EC: 2.3.2.13), which catalyzes lens crystallin cross-linking. Furthermore, studies show that supplementing with spermidine can delay lens opacification by replenishing spermidine levels and reducing lens crystallin cross-linking, which maintains lens transparency, suggesting its potential as a treatment for age-related cataracts [169,170].

Even though information about PA is limited for cataracts, these new findings hold promise for improving eye health, preventing vision loss, and potentially restoring sight, ultimately leading to a better quality of life.

In Table 1, we provide a summary of the benefits associated with the natural compounds previously discussed in relation to ARDs.

## 5. Natural Autophagy Activators in Other ARDs

The benefits of natural compounds that activate autophagy discussed in this review also extend to other ARDs such as osteoarthritis, sarcopenia, and type II diabetes (Table 2). We have described the involvement of key autophagic regulators, such as the Akt/mTOR, AMPK, FOXO3a and Sirt1, as the main effectors in enhancing the autophagic flux and ameliorating the consequences of these ARDs. These findings highlight the potential systemic health benefits that would bring new hope for ARDs.

Further research into the effects of natural compounds that stimulate autophagy could bring hope for better and healthier aging in fighting the associated diseases.

## 6. Conclusions

Age-related diseases such as cardiovascular, neurodegenerative, and ocular diseases represent a global social, economic, and health challenge [2]. Although the molecular mechanisms involved in aging and related diseases are not yet fully understood, it is known that processes like autophagy play a crucial role in the development of these pathologies. In fact, a decrease in the protein levels of key molecules in the autophagic flow, such as LC3 I/II, is one of their distinctive features [184]. As a result, various efforts have been made to propose new therapeutic targets that promote autophagic flow to restore cellular homeostasis in age-related diseases.

Our work focuses on compiling the most recent evidence regarding curcumin, resveratrol, polyamines, and quercetin as natural autophagy regulators for the treatment of age-related diseases. Natural molecular targets are of great interest due to their nutritional value and historical role as precursors in the development of new drugs. Molecules such as curcumin, quercetin, resveratrol, and some polyamines have been shown to enhance autophagy in both cellular and animal models [185]. Our work gathers recent evidence on the effects of administering these natural compounds that promote the stimulation of autophagy regulatory pathways, such as mTOR, FOXO1/3, AMPK, and Sirt1, leading to increased levels of key proteins such as Beclin-1 and LC3, as well as a decrease in p62. These effects of natural compounds have benefits on cardiovascular diseases, neurodegeneration, and cataracts [186]. Furthermore, other natural compounds like berberine have been shown to enhance autophagy AMPK and Sirt1 activation. Additionally, berberine is reported to extend lifespan and offer potential therapeutic benefits for ARDs such as sarcopenia and cardiovascular and neurodegenerative disorders [187,188,189,190,191,192].

To further explore the potential use of natural compounds as an adjuvant therapeutic strategy for ARDs, and given their better tolerability compared to other drugs, more research on long-term dosing is essential. For example, while curcumin and quercetin have been approved by the Food and Drug Administration (FDA) as “Generally Recognized As Safe” (GRS) [193,194], in clinical trials they have shown significant variability regarding dosage across different countries [195,196]. In contrast, evidence about resveratrol (8 mg/day) and spermidine (1.2 mg/day) did not seem to have major side effects in one-year and three-month clinical trials, respectively [197,198].

The combination of natural compounds with autophagy-enhancing drugs like rapamycin may be a promising strategy to fight ARDs due to their potential synergistic effects in promoting autophagy. However, there is currently insufficient evidence regarding these effects on ARDs. Most studies have primarily focused on anticancer therapies, where they have reported mechanisms of cell death via apoptosis or various forms of autophagy-related cell death [199,200,201,202]. However, natural compounds that stimulate autophagy also provide additional benefits, including anti-inflammatory and antioxidant properties, as well as improvements in mitochondrial dysfunction [59,203,204,205]. These cellular processes are closely linked to autophagy.

However, it is important to highlight that one of the main disadvantages of using natural compounds is their low bioavailability; thus, their effects may be mild. To address this, various efforts based on nanotechnology have been made to improve the bioavailability of these compounds. Other important factors to consider include the potential interactions with other drugs or supplements that could be harmful to health. Additionally, more experimental evidence is needed to support and confirm the benefits of curcumin, resveratrol, polyamines, and quercetin in age-related diseases.

## Figures and Tables

**Table 1:** Summary table of natural compounds and their autophagic effect on ARDs.

Age-Related Diseases	Natural Compound	Autophagic Effect	Ref.
Cardiopathies	Curcumin	Attenuation of oxidative stress and activation of autophagy via the AKT/mTOR regulation in diabetic rats with myocardial damage.	[52]
		Restoration of Beclin-1 and LC3 I/II ratio in mice with doxorubicin-induced cardiomyopathy.	[54]
	Resveratrol	Amelioration of cardiac dysfunction of neonatal rat ventricular myocytes via the negative regulation of the PI3K/Akt pathway and decreasing of apoptosis.	[66]
		Activation of TFEB for autophagy activation.	[67]
		Increased the expression of LC3 and Beclin-1 via Sirt1on endothelial oxidative damage of cardiovascular models.	[68]
	Quercetin	Activation of the AMPK pathway that leads to increased LC3 and Beclin-1 protein levels to ameliorate myocardial injury.	[79]
		Suppression of the PI3K/Akt pathway to attenuate myocardial ischemia.	[80]
		Prevention of myocardial fibrosis via the regulation of the miR-223/FOXO3 that leads to increasing LC3 II/I ratio.	[81,82]
	Polyamines	Prevention of lipidic plaque formation via increased expression of LC3 I/II ratio.	[94]
		Amelioration of myocardial dysfunction by promoting AMPK/mTOR pathway.	[95]
Alzheimer’s disease	Curcumin	Reverse cognitive disorders in an AD mice model induced by cisplatin by the activation of the AMPK-JNK signaling pathway to inhibit mTOR and Bcl-2, which modulates autophagy by the overexpression of LC3 and downregulation of p62.	[106]
	Resveratrol	Enhance cognitive function in rats by stimulating the AKT/mTOR pathway.	[109]
		Activation of the Sirt1 pathway, leading to increased mitophagy and subsequent neuroprotection.	[171]
		Improvements in sleep and memory dependent on the ortholog Sir2, a sirtuin ortholog that stimulates autophagy by activating HSP70 and ATG4 while it also interacts with LC3 and p62.	[111,112]
	Quercetin	Activates Sirt1 pathway, influencing the expression of FOXO1 and NRF2.	[118,119]
		Reduction of the Ab1 kinase expression, preventing tau phosphorylation and neuronal apoptosis triggered by Aβ.	[122,123]
		Research on a PC12 cell model showed quercetin improves cell survival and proliferation, mitigates Aβ toxicity, and enhances the expression of Sirt1.	[125,126]
	Polyamines	Elevates LC3 and Beclin-1 levels compared to control in SY5Y cells stably expressing a mutant form of human tau protein.	[90]
		Spermidines induce autophagy and clear amyloid precursor protein clusters.	[135]
Parkison’s disease	Curcumin	Increases the expression of LC3-II, facilitating enhanced clearance of α-synuclein proteins in mice models.	[107]
	Resveratrol	In mice model, ameliorates the deficit in cognitive and motor function in a dose-dependent manner along with a decrease in α-synuclein aggregation.	[114]
		In mice model, autophagic degradation of α-synuclein was promoted via the activation of the Sirt1 pathway.	[116]
		In two cell models, resveratrol promoted autophagy by increasing beclin-1 and the LC3-II/LC3-I ratio, and the reduction in p62.	[117]
	Quercetin	Ameliorates neuronal death, mitochondrial dysfunction, and α-synuclein accumulation in animal and cell models by activating autophagy.	[127]
		Increase of ULK1(Ser555)/ULK1, p-TBK1(Ser172)/TBK1, and LC3 levels.	[128]
		Increase autophagy and attenuate behavioral impairments.	[129]
	Polyamines	Autophagy stimulation	[90]
Ocular disorders	Curcumin	* Activation of TFEB to promote lysosomal biogenesis on rats with glucose-induced cataracts.	[149]
	Resveratrol	Increased expression of LC3 and Beclin-1 in high glucose treated HLEC cells.	[153]
	Polyamines	Downregulation of Sirt1 in age-related cataracts may be indirectly linked to altered polyamine (PA) levels in the human lens. Spermidine, known to positively regulate Sirt1, could play a role in this process.	[163,164]

* Curcumin analog C1.

**Table 2:** Natural autophagy activators in other ARDs.

Age-Related Disease	Natural Compound	Autophagic Effect	Ref.
Osteoarthritis	Curcumin Resveratrol Polyamines (Spermidine)	Reduction of miR-34a expression and probably through the Akt/mTOR pathway	[172]
		mTOR via the PI3K-Akt pathway	[172]
		Mitophagy AMPK/PINK1/Parkin pathway	[173]
		Activation of MAPK/ERK1/2 pathway	[174]
		AMPK/mTOR pathway	[175]
		Inhibition of CXCL16/ox-LDL pathway	[176]
		Overexpression of EP300 acetyltransferase	[177]
		Overexpression of BECN-1, LC3-II and p62	[178]
Sarcopenia	Resveratrol Polyamines (Spermidine)	SIRT1	[179]
		Probably AMPK-FOXO3a	[180]
Diabetes	Curcumin Polyamines (Spermidine)	In β cells miR-29b and miR-137 overexpression	[181]
		In podocyte via regulating Beclin1/UVRAG/Bcl2	[182]
		In podocyte regulate the AMPK/mTOR pathway	[183]

## Glossary

**Abbreviation**	**Meaning**
Abl	Abelson kinase
AD	Alzheimer’s disease
AKT	Alpha serine-threonine protein kinase
AMBRA1	Activating molecule in Beclin1-regulated autophagy protein 1
AMD	Age-related macular degeneration
AMPK	5′-adenosine monophosphate (AMP)-activated protein kinase
APP	Amyloid precursor protein
ARDs	Age-related diseases
Atg	Agy-related proteins
Aβ	Amyloid-β
Bcl-2	B-cell lymphoma 2 protein
BECN1	Beclin 1 gene
C1	curcumin analog
CAT	Catalase
CNS	Central nervous system
CXCL16	CXC motif chemokine ligand 16
EGFR	Epidermal growth factor
eIF5A	Initial Translate factor 5A Eukaryotic
EP300	Histone acetyltransferase P300
ERK	Extracellular signal-regulated kinase
FDA	Food and Drug Administration
FoxO	Forkhead box O proteins
HIIT	High intensity interval training
HLECs	Cultured lens epithelial cells
HSP70	70-kDa heat shock proteins
IGF	Insulin-like growth factor
JAK/STAT	Janus kinase-signal transducers and activators of transcription
JNK	c-jun-N-terminal kinase
LC3	Microtubule-associated protein 1A/1B-light chain 3 (LC3)
LV	Left ventricular
MAPK	Mitogen-activated protein kinase
MPP	1-methyl-4-phenylpyridium
MPTP	1-methyl-4-phenyl-1,2,3,6-tetrahydropyridine
MSVI	Severe vision impairment
mTOR	Mammalian target of rapamycin
NF-kB	Nuclear factor κB
NFTs	Neurofibrillary tangles
NMDA	N-methyl-D-aspartate
NRF2	Nuclear factor erythroid 2-related factor 2
Ox-LDL	Oxidized Low-Density lipoprotein
P62/SQSTM1	Sequestosome 1 (SQSTM1)/p62
PAs	Polyamines
PD	Parkinson´s disease
PdNPs	Palladium nanomaterial
PI3K	Phosphoinositide 3-kinase
PINK1	PTEN-induced putative kinase 1
PKA	Protein kinase A
p-TBK1	phospho-TANK-binding kinase 1
RPE	Retinal pigment epithelium
Sirt1	Silent information regulator 1
SNAP29	synaptosome associated protein 29
SNARE	Soluble N-ethylmaleimide-sensitive factor attachment protein receptor
SNCA	Synuclein alpha gene
SNHG1	small nucleolar RNA host gene 1
SOD	Superoxide dismutase
STX	Syntaxin 17
TBC1D20	TBC1 domain family, member 20
TFEB	Transcriptional Factor EB
THC	Tetrahydrocurcumin
TRAP	Translocon-associated protein
ULK	Ubiquitin-like-kinase
UPS	Ubiquitin-proteasome system
UVRAG	UV Radiation Resistance Associated
VAMP8	Vesicle associated membrane protein 8
VPS	Vacuolar protein sorting
